# A Deep Learning Framework for Evaluating the Over-the-Air Performance of the Antenna in Mobile Terminals

**DOI:** 10.3390/s24175646

**Published:** 2024-08-30

**Authors:** Yuming Chen, Dianyuan Qi, Lei Yang, Tongning Wu, Congsheng Li

**Affiliations:** China Academy of Information and Communications Technology, Beijing 100191, China; chenyuming314@outlook.com (Y.C.); qidianyuan@caict.ac.cn (D.Q.); yanglei@caict.ac.cn (L.Y.); wutongning@caict.ac.cn (T.W.)

**Keywords:** cellular phones, specific absorption rate, effective isotropic radiated power, deep learning

## Abstract

This study introduces RTEEMF (Real-Time Evaluation Electromagnetic Field)-PhoneAnts, a novel Deep Learning (DL) framework for the efficient evaluation of mobile phone antenna performance, addressing the time-consuming nature of traditional full-wave numerical simulations. The DL model, built on convolutional neural networks, uses the Near-field Electromagnetic Field (NEMF) distribution of a mobile phone antenna in free space to predict the Effective Isotropic Radiated Power (EIRP), Total Radiated Power (TRP), and Specific Absorption Rate (SAR) across various configurations. By converting antenna features and internal mobile phone components into near-field EMF distributions within a Huygens’ box, the model simplifies its input. A dataset of 7000 mobile phone models was used for training and evaluation. The model’s accuracy is validated using the Wilcoxon Signed Rank Test (WSR) for SAR and TRP, and the Feature Selection Validation Method (FSV) for EIRP. The proposed model achieves remarkable computational efficiency, approximately 2000-fold faster than full-wave simulations, and demonstrates generalization capabilities for different antenna types, various frequencies, and antenna positions. This makes it a valuable tool for practical research and development (R&D), offering a promising alternative to traditional electromagnetic field simulations. The study is publicly available on GitHub for further development and customization. Engineers can customize the model using their own datasets.

## 1. Introduction

Rapid and accurate assessment of the radiation performance and electromagnetic safety of mobile phones is crucial for RF antenna development and optimization. Traditional assessment methods employ full-wave numerical simulations for each R&D scenario, adhering to CTIA and IEC/IEEE requirements for over-the-air (OTA) and SAR evaluation [1,2,3,4]. The simulations need to employ the detailed Computer-Aided Design (CAD) models of the mobile phones with a commercial software package running on accelerated hardware. In most cases, simulations were conducted to select the appropriate antenna based on its performance within the device.

Nevertheless, this approach presents several challenges. First, the repetitive simulation of mobile phones is a time-consuming task. Evaluating the performance per frequency band should cover eleven configurations, including seven configurations for OTA (phone in free space; and both left and right ears in “cheek”, “data” mode, and “talk” mode) and four configurations for SAR (both left and right ears in “cheek” and “tilt”). Among them, ten configurations integrate the models of mobile phones, head, and hand in the numerical experiments. The calculation volume commonly comprises 10 M–200 M voxels, depending on the uniform or heterogeneous discretization strategy. A single simulation could take dozens of minutes depending on the acceleration hardware. The total time required for simulating all the possible configurations/frequencies can be enormous. Second, the preparation of the simulation scenarios is very labor-consuming. The standard rigorously defines the relative positioning of the mobile phone with the hand and head phantoms. Each joint of the hand phantom must deform precisely to achieve a correct holding posture, preventing any overlap between the hand and the device [5]. The manipulation can take up to ten hours with a specialized operator.

The aforementioned analysis necessitates reducing the number of numerical simulations in R&D, and/or reducing computational complexity for the individual simulation [6,7]. In this context, surrogate modeling has been applied in estimating the S11 of the antenna [8]. The method entails the generation of training instances (simulated results) through sampling within the parameter space (of the antenna’s geometry). As such, the relationship between the antenna dimensions and the S11 of the antenna was statistically regressed by polynomial expressions. This may indicate that the performance of the antenna can be theoretically predicted using the established model, thus eliminating the need for numerical simulations. The disadvantage is the curse of dimensionality, which may demand a large number of simulations for regressing the surrogate model. With the widespread application of DL techniques across diverse fields [9], their role in antenna design, optimization, and performance evaluation has emerged as a primary research interest [10]. The S parameters and the OTA results of the individual antenna (without the handset) have been estimated accurately. These studies suggest that we could take a step further by estimating the performance of the antenna using the end-to-end approach, to save computation time while reducing the number of simulations.

In practice, as the structure, geometry, and material of the specific antenna vary significantly, it is difficult to combine this information as the input for the DL models. The principle of the equivalent source (Huygens’ box [11]) could be a feasible approach. In such a case, we calculate the electric and magnetic current distribution induced by the antenna on a closed surface using the full-wave algorithm. The reconstructed electric and magnetic currents are then used as equivalent sources for EIRP and SAR evaluation [12]. It is demonstrated that the accuracy depends on the spatial resolution of the Huygens’ box. Specifically, the relative error of the calculation is less than 1% when the spatial resolution is less than 1/30 of the wavelength [13]. Therefore, the inputs to the DL model can be standardized by converting the geometry of the antennas into electromagnetic field (EMF) distributions on a Huygens’ box.

In the study, we proposed a DL framework for estimating the performance of the antenna installed on a mobile terminal. The model examines the near-field EMF distribution of a mobile phone antenna in free space (FS) and predicts EIRP and SAR for various configurations. To demonstrate the generalization of the proposed model, six types of commonly used patch antenna models [14] are positioned within a 6.1-inch mobile phone for evaluation. The DL network is trained with the simulated data of EIRP, TRP, and SAR across various configurations, resulting in a dataset of 7000 samples. The consistency of the results from the DL model and the full-wave simulation is demonstrated in the work.

The key contributions of this study are:

1. The proposed framework introduces a novel antenna performance prediction model, which can be used for preliminary assessment of SAR and EIRP while reducing the number of repetitive simulations with commercial software on the specialized computational platform;
2. The study converts the features of the antenna and the internal components of the mobile phone into a near-field EMF distribution on the Huygens’ box, which simplifies the input of the DL model;
3. A DL model based on the divide-and-conquer method [15] is proposed. It divides the estimation for SAR and EIRP into eight modules, reducing the training complexity and enhancing the convergence rate;
4. The Wilcoxon Signed Rank (WSR) test [16] for assessing SAR and TRP accuracy is developed. The Feature Selection Validation (FSV) [17] method is employed to evaluate EIRP accuracy. These methods provide a comprehensive understanding of the performance of the antenna.

## 2. The Proposed Method

### 2.1. The Workflow of the Proposed Method

**Figure 1:**
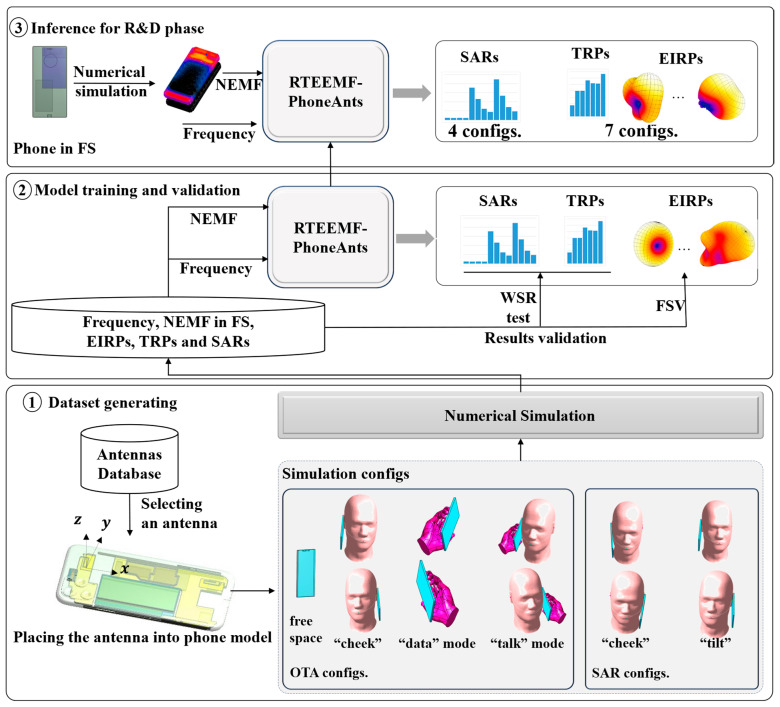
Workflow overview for the proposed method. This figure presents a detailed workflow for evaluating antenna performance, encompassing dataset generation, model training and validation, and inference during the R&D phase.

This study introduces an evaluation framework (RTEEMF-PhoneAnts) built upon convolutional neural networks. The operating frequency and Near Electromagnetic Field (NEMF) data of the mobile phone in FS serve as inputs for the model. Subsequently, the model predicts multiple performance metrics, including SAR, EIRP, and TRP for various configurations as required by [1,2,3,4]. The workflow is depicted in Figure 1.

Initially, an antenna database is developed, containing multiple patch antennas inserted into a simplified mobile phone model to simulate various of phone antenna configurations. Eleven configurations are established in accordance with [1,2,3,4]. Seven configurations are designated for EIRP and TRP evaluation, including:

- A phone in FS;
- A phone mounted in the “cheek” position on the head phantom (this includes the “cheek” position for both the left and right ears);
- PDA (Personal Digital Assistant) grip in “data” mode (for both the left and right hand);
- “Talk” mode (which includes configurations for the left and right head and hand).
- Additionally, four SAR evaluation configurations are created, including:
- The cheek (left and right) positions of the phone against the Specific Anthropomorphic Mannequin (SAM) phantom;
- Tilt (left and right) positions of the phone against the SAM phantom.

The positioning of the mobile phones relative to the SAM and hand phantoms strictly adheres to the requirements outlined in [1,2,3,4]. Subsequently, the Finite Difference Time Domain (FDTD) method is employed to evaluate the NEMF distribution for a phone in FS, as well as the EIRP, TRP, and SARs with a phone in these configurations. Following this, a dataset is generated containing the frequency, NEMF, EIRP, TRP, and SARs for use in model training and testing.

Secondly, a real-time antenna performance prediction model is developed and trained using the generated dataset. In this step, the mobile phone’s NEMF in FS serves as an input. Its distribution characteristics reflect the antenna’s radiation characteristics at the operating frequency as well as the influence of the mobile phone’s components on the antenna. The WSR test and FSV method are utilized to evaluate the model’s accuracy.

During practical R&D, the engineer simply calculates the NEMF for a new antenna in FS through numerical simulations. The NEMF and the operating frequency are input into the model, which then predicts the SARs for four configurations, and the EIRP and TRP for seven configurations. In this phase, there is no need to reconstruct simulation scenarios by precisely positioning the phone and manipulating the fingers as per [1,2,3,4], thereby further enhancing the evaluation efficiency.

### 2.2. RTEEMF-PhoneAnts Architecture

**Figure 2:**
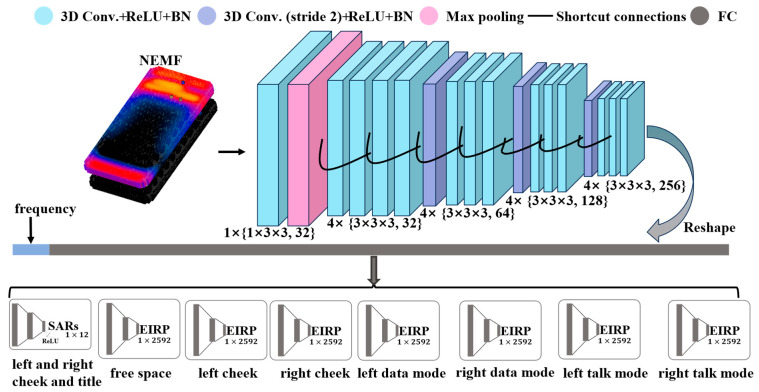
RTEEMF-PhoneAnts architecture.

The proposed model (RTEEMF-PhoneAnts) for predicting mobile phone antenna performance is depicted in Figure 2.

The model uses the NEMF of the mobile phone in free space and the operating frequency as inputs. A feature vector is extracted from the NEMF through a max pooling layer, multiple convolutional layers, and shortcut connections. Subsequently, the frequency is appended to the feature vector. To reduce the complexity of model training and accelerate the convergence, the divide-and-conquer method is introduced to process the SAR and EIRP estimation for eight modules. Each of them is constructed by three fully connected layers.

In the SAR prediction module, the Whole-Body Average SARs (WBSARs), the averaged peak SAR in 1 g and 10 g (SAR-1g and SAR-10g) tissue for four distinct scenarios, are output. The length of the output vector for the SAR prediction module is 1 × 12. The EIRP for seven scenarios is predicted using the prediction modules. Specifically, the EIRP prediction module provides output vectors with a resolution of 5° for both elevation angle (*θ*) and azimuth angle (*ϕ*). Consequently, the output vector length for the EIRP prediction module is 2592. TRP is computed from EIRP by

(1) $$\mathrm{TRP}=\frac{1}{4\pi }\int_{0}^{2\pi }\int_{0}^{\pi }\mathrm{EIRP}\left(\theta ,\phi \right)sin\theta d\theta d\phi .$$

## 3. Experiment Configurations

### 3.1. Dataset Generation

**Figure 3:**
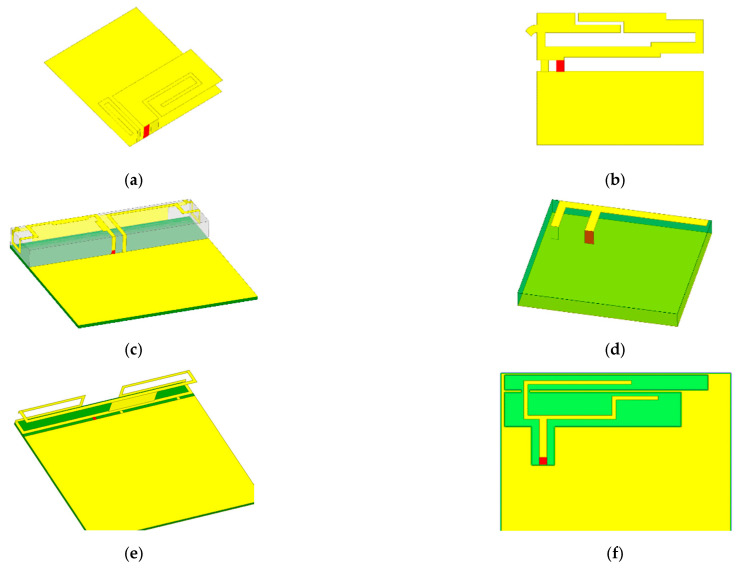
Digital models and operating frequencies of the six antennas developed in the study. (**a**) Ant. 1: Dual-band planar inverted-F(PlFA) with parasitic element. (**b**) Ant. 2: T-Branch gap Coupled Antenna. (**c**) Ant. 3: Folded Monopole Dipole Loop Antenna. (**d**) Ant. 4: Printed inverted-F antenna. (**e**) Ant. 5: loop antenna. (**f**) Ant. 6: dual-monopole slots antenna.

The study investigated six prevalent antenna types, covering the widely used bands from 2G to 5G wireless technology [14] (Figure 3). They are the most commonly used antenna types, including inverted-F antennas (IFAs) and printed IFAs (PIFAs) [18,19], monopole antennas [20] and monopole slot antennas [21], and loop antennas [22]. Among them, the PIFA and monopole are the most commonly used antenna types in mobile phones [23]. Comprehensive information about these antenna types is available in [18,19,20,21,22,23]. The operating principles of these antennas are well documented in [24,25].

For Ant. 1, Ant. 4, and Ant. 6, there are some overlaps in the frequency bands encompassed by antennas of various sizes, which are included to enrich the dataset. The parameters for Ant. 1–6 can be adjusted according to the methods described in [22,26,27,28].

**Figure 4:**
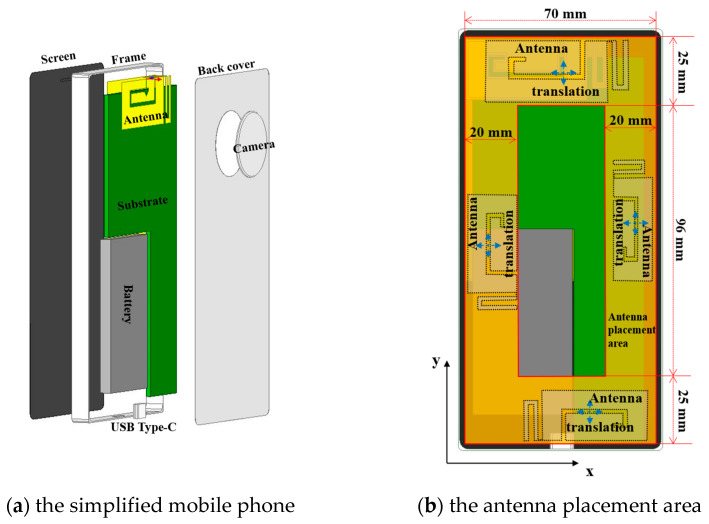
The simplified mobile phone model and the antenna placement regions. The antenna, battery, and USB Type-C are represented as perfect electric conductors. The relative permittivity (*ε_r_*) for the frame, screen, substrate, back cover, and camera are given as 3.8, 1.9, 4.2, 3.8, and 1.9, respectively.

A simplified 6.1-inch mobile phone model measuring 71.5 mm in width, 147.5 mm in length, and 7.85 mm in thickness, and consisting of eight components, is utilized (Figure 4a). Each antenna is positioned at various locations within the phone to simulate diverse antenna arrangements typical of real phones [14]. The top and bottom antennas are adjusted along the *x* and *y* axes in 1 mm increments, covering an area of 70 × 25 mm^2^, respectively. The side antenna is relocated along the *x* and *y* axes in 1 mm increments over an area of 146 × 20 mm^2^, respectively (Figure 4b). The distance between the antenna patch and the back cover remains fixed at 0.5 mm. Using Antenna Type Ant. 1 as an illustrative example, we selected ten antennas of varying dimensions within the Ant1 type. For each antenna, positions were sampled following the previously described position adjustment method. Ultimately, we sampled 240 distinct positions for each of the ten antennas (the number of sampling positions varied based on antenna structure and size). This process resulted in the creation of 2400 mobile phone models under the Ant1 type (Table 1). Ultimately, 7000 mobile phone models featuring various antenna configurations were produced.

The simulation is conducted using SEMCAD V19.2 (SPEAG AG, Zurich, Switzerland), employing the FDTD algorithm. Adaptive non-uniform grids are utilized to preserve antenna details in the discretization process. The maximum grid step size is set at 2 mm, with a non-uniform edge ratio maintained at no more than 1.2. The calculations were performed over 30 periods. Dielectric parameters, from references [1,4], are applied to the SAM and hand phantoms. When the phone is in FS, a Huygens’ box measuring 72 × 148 × 7.9 mm^3^ surrounding the phone is established to record the NEMF. The results were generated and exported for the NEMF in FS (complex (which include magnitude and phase) vector field $E_{x},E_{y},E_{z}$ and $H_{x},H_{y},H_{z}$ with a spatial interval of 0.5 × 0.5 × 0.1 mm^3^), as well as the SARs (WBSAR, SAR-1g, SAR-10g), TRP, and EIRP (angular resolution of 5°) in various configurations. All simulation results have been normalized to a 1 W input power.

The numerical simulations for EMF were conducted using a workstation with two Intel Xeon Gold 6230 CPUs (Intel, Santa Clara, USA) @ 2.1 GHz, 64 GB RAM, and two Quadro P5000 GPUs (NVIDIA, Santa Clara, USA; 32 GB memory on board).

### 3.2. Data Preprocessing for RTEEMF-PhoneAnts

The normalization and standardization methods are employed to enhance the convergence of the model [29]. In this study, the frequency normalization process is executed as follows:

(2) $$fre^{\prime }=\frac{fre_{MHz}-600}{6000-600},$$

where $fre_{MHz}$ represents the operational frequency in MHz.

SAR and OTA performance standardization process is performed as follows:

(3) $$x^{\prime }=\frac{x-\mu }{\sigma },$$

where x represents SARs, TRP, and EIRP for various configurations, μ denotes the population mean, and σ indicates the standard deviation.

The NEMF is uniformly resampled into an array with a spatial resolution of 0.5 × 0.5 × 0.1 mm^3^, 1 × 1 × 0.2 mm^3^, and 2 × 2 × 0.5 mm^3^. Correspondingly, the model inputs for these resolutions are 143 × 295 × 79, 72 × 148 × 40, and 36 × 74 × 16, respectively.

### 3.3. Training Configurations for RTEEMF-PhoneAnts

The workstation is equipped with two Intel^®^ Xeon^®^ Gold 5218 CPUs and four NVIDIA V100 GPUs, which are utilized for model training and testing. The optimized hyperparameters are as follows: a batch size of 4, a base learning rate of 0.02, the use of the Adam optimizer, and a maximum of 200 iterations.

### 3.4. Evaluation Criteria and Numerical Validation

The Root Mean Squared Error (RMSE) is utilized as the loss function to optimize network parameters.

(4) $$loss_{x}=\sqrt{\frac{1}{N}\sum_{i=1}^{N}{\left(y_{i}-{\hat{y}}_{i}\right)}^{2}},$$

where $y_{i}$ signifies standardized simulation results for SARs, TRP, and EIRP, while ${\hat{y}}_{i}$ represents the model’s predicted results for these metrics. The comprehensive loss function is articulated as follows:

(5) $$Loss=Loss_{SAR}+Loss_{EIRP}.$$

The SAR component of the loss is calculated using

(6) $$Loss_{SAR}=\frac{\sum_{i=1}^{4}{\left(loss_{WBSAR}+loss_{SAR-1g}+loss_{SAR-10g}\right)}_{i}}{12},$$

where i represents the four configurations of the SAR test.

The EIRP component of the loss is calculated using

(7) $$Loss_{EIRP}=\frac{\sum_{i=1}^{7}{\left(loss_{EIRP}\right)}_{i}}{7},$$

where i represents the seven configurations of the OTA test.

To evaluate the model’s accuracy, model inference outcomes were compared with simulation results. The WSR test [16] was used to determine the model’s precision in SAR and TRP metrics. FSV [17] was applied to gauge the EIRP’s accuracy. The analyses were performed using SPSS 19.0 (SPSS Inc., Chicago, IL, USA) and Matlab 2019 (MathWorks, Apple Hill Drive, Natick, MA, USA).

Stratified Sampling (SS) and the Leave-One-Antenna-Out (LOAO) approach were used to verify the model’s generalizability. SS required a random partition of each antenna category into training and testing sets in a 9:1 ratio. In contrast, the LOAO approach involved training on five antennas, while setting aside one antenna exclusively for testing. The SS method can demonstrate the model’s generalization performance across diverse frequencies and antenna positions. Conversely, the LOAO method is capable of evaluating the model’s generalization across various antenna types.

## 4. Experiment Results

### 4.1. Performance of the Proposed Model

**Figure 5:**
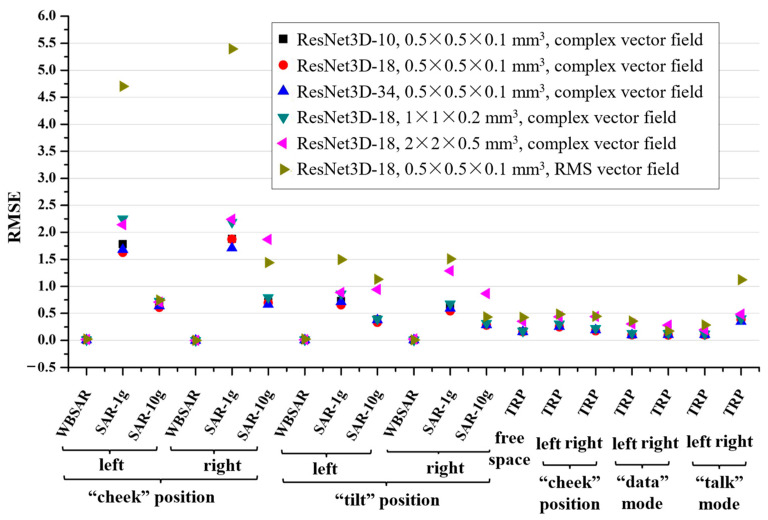
The effects of varying model depths, spatial resolutions, and NEMF types on the RMSE of the predicted outcomes.

To determine the most effective configuration for our approach, the dataset was partitioned into training and test sets using Stratified Sampling (SS). The model’s accuracy was influenced by various factors, including the ResNet3D model depth, NEMF spatial resolution, and data formats (complex (magnitude and phase) vector field and Root Mean Square (RMS) vector field). Figure 5 illustrates the average RMSE on the test dataset, reflecting the impact of these factors.

Initially, complex NEMF, with a resolution of 0.5 × 0.5 × 0.1 mm^3^ was input into three models of varying depths (illustrated by black squares, red circles, and blue triangles in Figure 5). The RMSEs for WBSAR and TRP predictions showed minor variations across different model depths, with ResNet3D-18 yielding the most accurate results for SAR-1g and SAR-10g.

Subsequently, ResNet3D-18 served as the backbone network, and the impacts of three distinct NEMF spatial resolutions (indicated by red circles, green inverted triangles, and pink left triangles in Figure 5) were compared. The analysis revealed that NEMF resolutions of 1 × 1 × 0.2 mm^3^ and 2 × 2 × 0.5 mm^3^ resulted in higher RMSEs for SARs and TRP.

Finally, when applying a resolution of 0.5 × 0.5 × 0.1 mm^3^ for both complex and RMS NEMF (represented by red circles and brass right triangles in Figure 5) with ResNet3D-18 as the framework, a significant discrepancy in SAR-1g from the reference value was observed using RMS NEMF as the input.

The WSR test reveals no significant differences (*p* > 0.05) between the predicted SAR and TRP and the SEMCAD results (Table 2), highlighting the proposed methodology’s consistency with the established methods.

**Figure 6:**
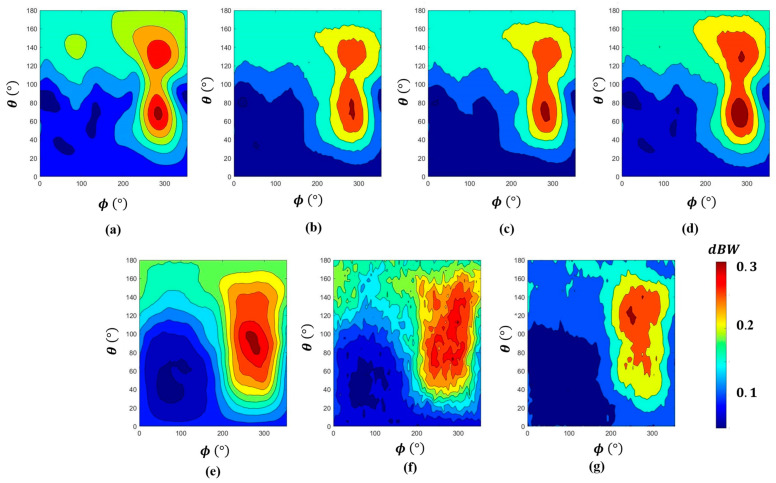
A filled contour plot of EIRP prediction using complex and RMS NEMF at various resolutions and different depths of ResNet3D networks. (**a**) Reference from SEMCAD. (**b**) ResNet3D-10 0.5 × 0.5 × 0.1 mm^3^ Complex vector field. (**c**) ResNet3D-18 0.5 × 0.5 × 0.1 mm^3^ Complex vector field. (**d**) ResNet3D-34 0.5 × 0.5 × 0.1 mm^3^ Complex vector field. (**e**) ResNet3D-10 0.5 × 0.5 × 0.1 mm^3^ RMS vector field. (**f**) ResNet3D-10 1 × 1 × 0.2 mm^3^ Complex vector field. (**g**) ResNet3D-10 2 × 2 × 0.5 mm^3^ Complex vector field. ***ϕ*** represents the azimuth angle, ***θ*** represents the elevation angle.

Figure 6 shows the predicted EIRPs from diverse model configurations. EIRP distributions estimated by the model utilizing RMS NEMF or a coarser spatial resolution exhibit notable variance from the SEMCAD results. In contrast, greater consistency in distribution is observed with the SEMCAD results when employing the finest spatial resolution and complex NEMF with ResNet3D-34.

Figure 6 shows that the regions with higher EIRP values, depicted in deep red, represent the main lobe of the EIRP. The results from Figure 6b–d demonstrate good agreement with the simulation results obtained using the commercial software SEMCAD V19.2, accurately reflecting the main lobe. Notably, the highest consistency in distribution with the SEMCAD results is achieved when employing the finest spatial resolution and the complex NEMF with ResNet3D-34, as shown in Figure 6d.

**Figure 7:**
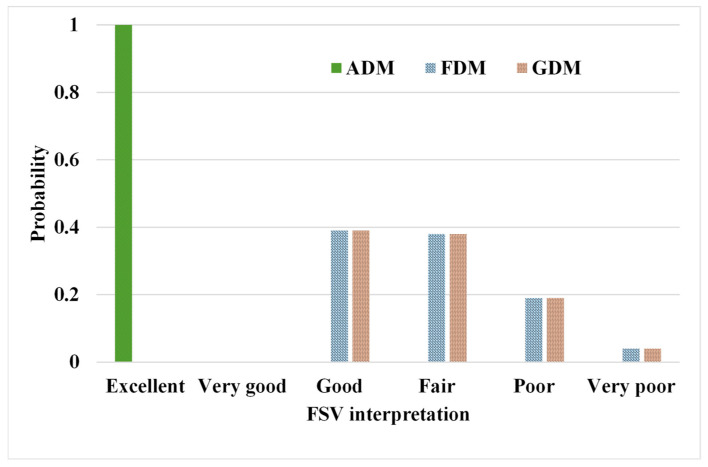
Histogram illustrating the confidence levels of FSV, ADM, FDM, and GDM results in model predictions as compared to SEMCAD.

The precision of the predicted EIRP was evaluated via FSV (Figure 7), demonstrating “excellent” amplitude concordance [30] between the proposed model and SEMCAD results as per ADM (amplitude difference measure). In contrast, the FDM (Feature Difference Measure) and GDM (Global Difference Measure) reveal broader probability density functions, indicating variability in findings between the proposed model and SEMCAD results, thereby underscoring challenges in predicting detailed local EIRP [31]. This inconsistency arises from the absence of constraints on adjacent elements in the terminal fully connected layer of the EIRP prediction module. Implementing smoothing constraints after the EIRP module could ameliorate this issue.

### 4.2. Efficiency of the Proposed Model

In terms of computational efficiency, SEMCAD assessed antenna configurations at a specific frequency, without accounting for the time that the simulation engineers invest in configuring scenarios. FDTD analysis requires approximately 5 h per scenario (4 SAR configurations plus 7 EIRP configurations) using the accelerating hardware and the commercialized software, whereas the proposed model processes all scenarios in less than 3 s using the proposed method. Thus, simulation resources are significantly saved. As shown in Table 3, the proposed model significantly reduces the time cost compared to the full-wave simulation method.

Typically, a mobile phone antenna can accommodate multiple frequency bands. According to the 3GPP TS 36.101 standard [32,33], User Equipment (UE) can have up to 90 Uplink (UL) operating frequency bands. Commercially available mobile phones typically feature multi-antenna configurations, often with ten or more antennas [34].

**Figure 8:**
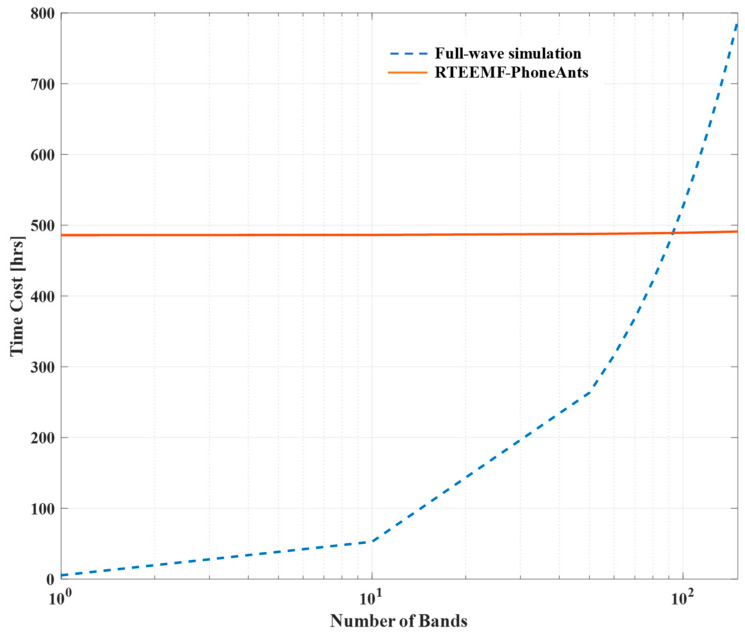
Comparison of computational time between RTEEMF-PhoneAnts and full-wave simulation as the number of frequency bands increases.

The full-wave simulation technique is used for simulations, each of which corresponds to an individual frequency band of a mobile phone. As illustrated in Figure 8, when this technique is used to simulate more than approximately 100 frequency bands, the time consumption gradually surpasses the time required to train a DL model. Concurrently, the prediction time of the DL model is approximately 2000 times faster than that of the full-wave simulation method. Therefore, as the number of frequency bands simulated increases, the time efficiency advantage of the DL model also increases correspondingly.

### 4.3. Model Generalization across Diverse Antenna Types

Table 4 details the average deviation values and their corresponding standard deviations. The elevated average deviation associated with the Leave-One-Antenna-Out (LOAO) method compared to the Stratified Sampling (SS) method implies a significant influence of the antenna type on the model ability to generalize. Both the SS and LOAO methods exhibit an average deviation (%) within the range of 8% and 13%, and a maximum deviation within 13% and 25%, respectively. When the LOAO method is applied with Antenna 2 and Antenna 3 designated as test sets, the deviation decreases, with an average deviation within 9% and a maximum deviation within 18%.

IEC/IEEE 62209-1528 [4] stipulates that the uncertainty in SAR testing should be less than 30%. Concurrently, the OTA measurement standard [1] asserts that the uncertainty in Free Space (FS) measurements should be less than 2 dB (approximately 58.5%), while the uncertainty requirements for the aforementioned six other configurations are more lenient (2.2–2.4 dB). This suggests that our model demonstrates generalization capabilities for different antenna types, varying frequencies, and antenna positions.

Additionally, the deviations are reduced for Ant.2 and Ant.3 when designated as test sets, potentially due to the narrower sampling intervals in the training dataset, which facilitate the assembly of more extensive training sets (Table 1). Conversely, selecting Ant.1 and Ant.6 as test sets yields elevated average deviation compared to other validation frameworks. This increase is tentatively linked to a paucity of data, which tends to entrap the model in a state of overfitting. Hence, it is critical to augment the volume of training data.

**Figure 9:**
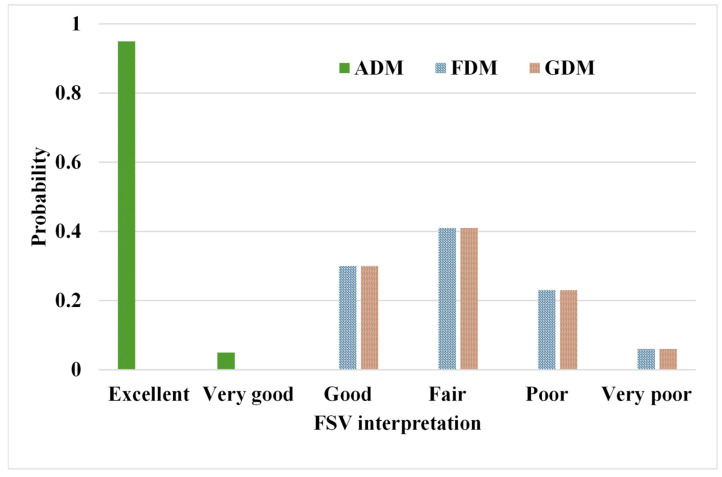
Confidence histogram of FSV, ADM, FDM, and GDM results for the model prediction with respect to the SEMCAD when Ant.6 is used as a test set.

As discerned from Table 4, Antenna 6 exhibits the greatest deviation when compared to the simulation results. Consequently, it was used as a test set in the study, and the consistency of the model predictions with the SEMCAD simulation results was assessed using the WRS test and FSV, as shown in Table 5 and Figure 9, respectively.

Firstly, Table 5 shows that there is no significant difference (*p* > 0.05) in the SAR and OTA performance for Ant.6 when compared to the SEMCAD and model predictions. Secondly, the FSV presented in Figure 9 shows that the magnitude of the EIRP predicted by the model is in good agreement with the SEMCAD results (ADM exhibits “Excellent” and “Very good”). However, more than 30% of the predictions of the FDM and GDM exhibit “Poor” or “Very poor” results. This suggests that improvements are needed for the predictions of the EIRP details.

Admittedly, the proposed framework aims to provide an alternative method for estimating antenna performance while saving the simulation time in R&D. Hence, the generated DL model does not aim to provide a versatile solution for estimating the performance of each antenna. The dimensions and material of the mobile phones were not considered as the variables in the study. This is feasible because the dimensions and materials are convergent for the current mobile phones. For example, a survey performed by UBI Research revealed that a total of 122 OLED (Organic Light-Emitting Diode)-screen handsets were launched between January and June of 2021, and of those, 97.5% (or 119, to be exact) were in the 6-inch size range. In addition, a recent study [35] revealed no significant statistical differences between groups of smartphones based on their construction materials. As mentioned previously, the types of antennas used in current mobile phones are limited [14]. Therefore, the results are representative, and the framework we proposed is applicable to R&D.

We have established that a spatial resolution of less than 1/30 of the wavelength is instrumental in maintaining a relative error below 1% [13]. This finding has significant implications for the standardization of inputs to our DL model. By transforming the antenna geometry into NEMF distributions on a Huygens’ box, we can streamline the input process for the DL model.

This study presents several limitations. Firstly, the transferability of the model could be compromised by the constrained scope of antenna types and configurations evaluated, encompassing only six prevalent antenna patterns. Secondly, the use of a streamlined mobile model disregarded the intricacies and heterogeneity of materials found in genuine phone components, which may result in divergences when assessing authentic antenna functionality.

## 5. Conclusions

We have developed a DL framework for evaluating mobile phone antenna performance, utilizing operating frequency and NEMF to estimate SAR and OTA performance with notable precision across various configurations. This framework enables preliminary assessment of SAR and EIRP while significantly reducing the need for repetitive simulations using specialized computational software. By leveraging DL, we streamline the evaluation process, making it more efficient and accessible. We convert antenna features and internal mobile phone components into near-field EMF distributions within a Huygens’ box. This simplifies input for the DL model. Our DL model adopts a divide-and-conquer approach, splitting SAR and EIRP estimation into eight modules. This reduces training complexity and improves convergence rates. Compared to full-wave simulations, our proposed model achieves remarkable computational efficiency. It processes all scenarios in seconds, making it a valuable tool for practical R&D.

We use the WSR test for SAR and TRP accuracy evaluation. Additionally, FSV assesses EIRP accuracy. While our model demonstrates excellent agreement with SEMCAD results, challenges remain in predicting detailed local EIRP due to the lack of constraints on adjacent elements in the terminal fully connected layer. Despite these limitations, our framework provides a valuable alternative to time-intensive simulations. To enhance its applicability, future work should expand the dataset to include additional antenna types and configurations, as well as more realistic mobile models.

In summary, our DL framework provides an efficient means of assessing mobile phone antenna performance. Future work should address model transferability and consider material heterogeneity for more accurate evaluations. Overall, our study is accessible on GitHub for free use (https://github.com/licongsheng/rteemf-phoneants, accessed on 10 August 2024). Engineers can customize the model using their own datasets, presenting a viable substitute for traditional electromagnetic field simulations.

## Figures and Tables

**Table 1:** Operating frequencies supported by the antennas used in the study.

fre (MHz)	Bands	Ant.					
		1	2	3	4	5	6
850	GSM850,WCDMA	√	√	√	√	√	√
900	GSM900,UMTS900	√	√	√	√	√	√
1800	GSM1800, WCDMA,FDD-LTE	√	√	√	√	√	√
1900	GSM1900,TD-SCDMA,TDD-LTE	√	√	√	√	√	√
2000	TD-SCDMA	√	√		√	√	√
2100	WCDMA,CDMA2000	√	√	√	√	√	√
2600	TDD-LTE	√			√		√
Number of antennas		10	1	1	7	1	14
Number of samples		2400	200	220	1600	180	2400

The symbol “√” indicates that the corresponding antenna supports the specified operating frequency band.

**Table 2:** Comparison of the results by the model proposed in this study and SEMCAD.

Configurations			25th Percentile		75th percentile		Z-Value	*p*-Value
			SEMCAD	RTEEMF-PhoneAnts	SEMCAD	RTEEMF-PhoneAnts		
“cheek” position (W/kg)	left	WBSAR	0.05	0.05	0.13	0.12	−0.20	0.84
		SAR-1g	6.35	6.74	20.43	20.84	−0.06	0.95
		SAR-10g	1.81	1.79	13.66	13.78	−1.77	0.08
	right	WBSAR	0.05	0.05	0.13	0.13	−0.71	0.48
		SAR-1g	14.50	13.31	38.40	40.35	−0.20	0.84
		SAR-10g	7.70	7.40	16.99	16.65	−0.22	0.83
“tilt” position (W/kg)	left	WBSAR	0.06	0.06	0.13	0.13	−0.20	0.84
		SAR-1g	2.18	2.26	13.89	15.11	−0.59	0.56
		SAR-10g	1.27	1.27	5.80	5.92	−1.07	0.29
	right	WBSAR	0.06	0.06	0.13	0.12	−0.82	0.41
		SAR-1g	7.92	7.41	23.37	20.89	0.00	1.00
		SAR-10g	2.15	2.03	8.30	8.05	−0.47	0.64
free space (dB)		TRP	2.53	1.69	3.56	3.91	0.00	1.00
“cheek” position (dB)	left	TRP	−6.02	−5.04	−0.08	−0.04	0.00	1.00
	right	TRP	−5.59	−5.42	0.10	0.11	−1.37	0.17
“data” mode (dB)	left	TRP	0.32	0.18	1.65	1.55	−1.77	0.08
	right	TRP	−0.36	−0.33	1.80	1.96	0.00	1.00
“talk” mode (dB)	left	TRP	−0.34	−0.34	2.00	1.95	−0.63	0.53
	right	TRP	−8.70	−8.43	−4.37	−2.93	−1.37	0.17

**Table 3:** Comparison of time cost by the proposed model and full-wave simulation method.

Configurations		Time Cost (min)			
		Full-Wave Simulation for One Band		RTEEMF-PhoneAnts	
		Position of Phone	Simulation	Training (Number of Training Data = 7000, Batch Size = 4, Epoch = 200)	Inference (Batch Size = 1)
SAR evaluation	Left cheek	0.5	20	29,167	2.025
	Left tilt	2	24		
	Right cheek	0.5	20		
	Right tilt	2	24		
OTA evaluation	Phone in free space	0	2		
	Left cheek	0	0		
	Right cheek	0	0		
	Left hand in “data” mode	3	30		
	Right hand in “data” mode	3	30		
	Left hand and head in “talk” mode	4	65		
	Right hand and head in “talk” mode	4	65		

**Table 4:** Comparison between the model proposed in this study and the SEMCAD calculation results.

Configurations			Mean ± Std of Deviation (%), $\mathrm{Deviation}=\frac{\left(\mathrm{Simulation}-\mathrm{DL}\right)}{\mathrm{Simulation}}\times 100\%$						
			Stratified Sampling	Leave-One-Antenna-Out					
				Ant.1	Ant.2	Ant.3	Ant.4	Ant.5	Ant.6
“cheek” position	left	WBSAR	5.84 ± 2.52	6.35 ± 4.25	7.82 ± 4.61	4.97 ± 4.07	9.5 ± 3.66	8.51 ± 3.58	9.06 ± 3.94
		SAR-1g	5.71 ± 2.84	5.99 ± 5.59	5.98 ± 3.87	5.75 ± 3.36	8.25 ± 3.14	5.76 ± 4.52	8.85 ± 3.61
		SAR-10g	3.71 ± 0.28	5.3 ± 0.43	3.73 ± 0.46	3.71 ± 0.5	4.33 ± 0.56	4.58 ± 0.39	5.76 ± 0.55
	right	WBSAR	4.93 ± 2.29	6.63 ± 4.04	5.09 ± 3.05	5.54 ± 2.74	8.74 ± 2.95	5.26 ± 3.41	7.64 ± 4.25
		SAR-1g	2.15 ± 0.75	2.5 ± 1.09	3.61 ± 1.18	2.68 ± 1.12	3.92 ± 1.19	3.61 ± 1.02	3.34 ± 0.77
		SAR-10g	3.22 ± 1.88	3.82 ± 2.81	3.24 ± 2.25	3.7 ± 2.09	5.7 ± 3.76	4.36 ± 2.53	4.99 ± 2.99
“tilt” position	left	WBSAR	5.26 ± 2.63	6.07 ± 2.84	7.36 ± 4.21	7.76 ± 4.72	9.69 ± 4.82	7.51 ± 3.59	8.16 ± 3.61
		SAR-1g	7.17 ± 3.97	10.5 ± 4.67	7.35 ± 5.48	7.28 ± 4.23	9.98 ± 5.25	7.71 ± 7.8	11.11 ± 4.69
		SAR-10g	6.04 ± 2.96	7.97 ± 4.7	7.35 ± 3.52	6.97 ± 4.15	10.86 ± 4.6	7.39 ± 4.29	9.36 ± 3.32
	right	WBSAR	3.46 ± 1.83	4.61 ± 2.93	5.57 ± 2.51	4.03 ± 2.23	4.35 ± 1.97	4.21 ± 3.27	5.36 ± 2.93
		SAR-1g	6.09 ± 2.88	8.43 ± 4.97	8.21 ± 3.9	8.31 ± 4.13	9.79 ± 4.1	7.21 ± 5.03	9.44 ± 3.22
		SAR-10g	5.07 ± 2.8	6.32 ± 4.25	6.22 ± 5.24	6.78 ± 3.37	7.86 ± 5.49	6.67 ± 2.91	7.86 ± 4.48
free space		TRP	0.24±0.12	1.91 ± 0.93	2.49 ± 1.16	2.97 ± 1.04	2.15 ± 1.31	2.1 ± 1.03	3.32 ± 1.56
“cheek” position	left	TRP	3.92 ± 1.16	5.26 ± 1.82	5.24 ± 1.95	4.09 ± 2.05	6.92 ± 2.32	4.08 ± 2.05	6.08 ± 2.24
	right	TRP	3.48 ± 1.55	4.78 ± 3.08	3.75 ± 2.66	4.76 ± 2.24	4.8 ± 2.22	4.47 ± 1.67	5.4 ± 2.2
“data” mode	left	TRP	1.9 ± 1.18	2.22 ± 2.17	3.05 ± 2.11	2.8 ± 1.22	3.39 ± 2.05	2.97 ± 1.58	2.95 ± 1.78
	right	TRP	1.81 ± 0.82	2.35 ± 1.53	2.83 ± 1.45	2.13 ± 1.41	2.06 ± 1.4	2.09 ± 1.29	2.8 ± 1.09
“talk” mode	left	TRP	2.87 ± 1.37	3.71 ± 1.73	2.98 ± 2.35	3.05 ± 1.46	3.43 ± 2.17	3.89 ± 2.09	4.44 ± 2.55
	right	TRP	7.88 ± 4.49	9.41 ± 8.72	8.79 ± 6.11	8.54 ± 6.72	9.83 ± 7.93	7.93 ± 8.16	12.21 ± 8.4

**Table 5:** Comparison between the model proposed in this study and the SEMCAD calculation results.

Configurations			25th Percentile		75th Percentile		Z-Value	*p*-Value
			SEMCAD	RTEEMF-PhoneAnts	SEMCAD	RTEEMF-PhoneAnts		
“cheek” position (W/kg)	left	WBSAR	0.04	0.05	0.13	0.13	−1.34	0.18
		SAR-1g	13.05	13.34	41.37	42.90	−0.42	0.68
		SAR-10g	5.92	5.97	18.50	18.06	−0.39	0.69
	right	WBSAR	0.02	0.02	0.08	0.08	−0.77	0.44
		SAR-1g	1.75	1.71	13.88	13.57	−0.67	0.50
		SAR-10g	1.13	1.20	7.97	7.67	−0.22	0.83
“tilt” position (W/kg)	left	WBSAR	0.05	0.06	0.13	0.13	−0.72	0.47
		SAR-1g	13.56	13.55	35.55	34.76	−0.98	0.33
		SAR-10g	7.36	7.54	17.04	18.10	−2.37	0.02
	right	WBSAR	0.02	0.02	0.08	0.08	−1.13	0.26
		SAR-1g	2.27	2.16	14.37	14.24	−0.38	0.70
		SAR-10g	1.27	1.34	8.18	7.61	−0.44	0.66
free space (dB)		TRP	2.99	2.92	3.60	3.73	−0.04	0.97
“cheek” position (dB)	left	TRP	−6.43	−6.89	−0.09	−0.09	−0.05	0.96
	right	TRP	−5.95	−5.37	0.11	0.11	−1.66	0.10
“data” mode (dB)	left	TRP	0.37	0.32	1.72	1.79	−1.17	0.24
	right	TRP	−0.35	−0.34	2.40	2.36	−1.09	0.28
“talk” mode (dB)	left	TRP	−0.33	−0.34	2.36	2.25	−0.17	0.86
	right	TRP	4.64	4.43	9.11	8.02	−0.16	0.87

## Data Availability

The original contributions presented in the study are included in the article; further inquiries can be directed to the corresponding author.

## Abbreviations

The following abbreviations are used in this manuscript:

RTEEMF-PhoneAnts	Real-Time Evaluation Electromagnetic Field–Phone Antennas	DL	Deep Learning
NEMF	Near-field Electromagnetic Field	EMF	Electromagnetic Field
EIRP	Effective Isotropic Radiated Power	TRP	Total Radiated Power
SAR	Specific Absorption Rate	WBSAR	Whole-Body Average SAR
SAR-1g	averaged peak SAR in 1g	SAR-10g	averaged peak SAR in 10g
WSR	Wilcoxon Signed Rank Test	FSV	Feature Selection Validation Method
R&D	Research and Development	CTIA	Cellular Telecommunications and Internet Association
IEC	International Electrotechnical Commission	IEEE	Institute of Electrical and Electronics Engineers Engineers
CAD	Computer-Aided Design	FS	Free Space
FDTD	Finite Difference Time Domain	PDA	Personal Digital Assistant
SAM	Specific Anthropomorphic Mannequin	IFAs	Inverted-F Antennas
PIFAs	Printed IFAs	Ant	Antenna
RMSE	Root Mean Squared Error	RMS	Root Mean Square
SS	Stratified Sampling	LOAO	Leave-One-Antenna-Out
ADM	Amplitude Difference Measure	FDM	Feature Difference Measure
GDM	Global Difference Measure	3GPP	3rd Generation Partnership Project
UE	User Equipment	UL	Uplink
OLED	Organic Light-Emitting Diode		

## References

[1] CTIA Certification (2019). Test Plan for Wireless Device Over-the-Air Performance: Method of Measurement for Radiated RF Power and Receiver Performance.

[2] (2017). Determining the Peak Spatial-Average Specific Absorption Rate (SAR) in the Human Body from Wireless Communications Devices, 30 MHz to 6 GHz—Part 1: General Requirements for Using the Finite-Difference Time-Domain (FDTD) Method for SAR Calculations.

[3] (2017). Determining the Peak Spatial-Average Specific Absorption Rate (SAR) in the Human Body from Wireless Communications Devices, 30 MHz to 6 GHz—Part 2: Specific Requirements for Finite Difference Time Domain (FDTD) Modelling of Exposure from Vehicle Mounted Antennas.

[4] (2020). Measurement Procedure for the Assessment of Specific Absorption Rate of Human Exposure to Radio Frequency Fields from Hand-Held and Body-Mounted Wireless Communication Devices—Part 1528: Human Models, Instrumentation, and Procedures (Frequency Range of 4 MHz to 10 GHz).

[5] Li C., Yang L., Wu T. (2021). Predicting Over-the-Air Performance Using a Hand Phantom with Large Dielectric Flexibility. IEEE Trans. Electromagn. Compat..

[6] Li C., Wu T. (2023). Efficient Evaluation of Incident Power Density by Millimeter-Wave MIMO User Equipment Using Vectorized Field Superposition and Stochastic Population Optimizers. IEEE Trans. Electromagn. Compat..

[7] Wu T., Chen Y., Li C. (2024). Efficient Evaluation of Epithelial/Absorbed Power Density by Multi-Antenna User Equipment with SAM Head Model. IEEE Antennas Wirel. Propag. Lett..

[8] Koziel S., Dabrowska A.P. (2019). Performance-Based Nested Surrogate Modeling of Antenna Input Characteristics. IEEE Trans. Antenn. Propag..

[9] Zhai M., Chen Y., Xu L., Yin W.Y. (2023). An End-to-End Neural Network for Complex Electromagnetic Simulations. IEEE Antennas Wirel. Propag. Lett..

[10] Khan M.M., Hossain S., Mozumda P., Akte S., Ashique R.H. (2022). A review on machine learning and deep learning for various antenna design applications. Heliyon.

[11] Love A. (1901). The integration of the equations of propagation of electric waves. Phil. Trans. Roy. Soc. London Ser. A.

[12] Scialacqua L., Mioc F., Scannavini A., Foged L.J. Simulated and Measured Power Density using Equivalent Currents for 5G applications. Proceedings of the 2020 IEEE International Symposium on Antennas and Propagation and North American Radio Science Meeting.

[13] Eibert T.F., Kiliç E., Lopez C., Mauermayer R.A., Neitz O., Schnattinger G. (2015). Electromagnetic Field Transformations for Measurements and Simulations (Invited Paper). Prog. Electromagn. Res..

[14] Wang H. (2022). Overview of Future Antenna Design for Mobile Terminals. Engineering.

[15] Li C., Yao G., Xu X., Yang L., Zhang Y., Wu T., Sun J. (2020). DCSegNet: Deep Learning Framework Based on Divide-and-Conquer Method for Liver Segmentation. IEEE Access.

[16] Noether G.E. (1992). Introduction to Wilcoxon (1945) individual comparisons by ranking methods. Breakthroughs in Statistics: Methodology and Distribution.

[17] Duffy A.P., Martin A.J., Orlandi A., Antonini G., Benson T.M., Woolfson M.S. (2006). Feature selective validation (FSV) for validation of computational electromagnetics (CEM). part I-the FSV method. IEEE Trans. Electromagn. Compat..

[18] Liu Z., Hall P.S., Wake D. (1997). Dual-frequency planar inverted-F antenna. IEEE Trans. Antenn. Propag..

[19] Khlouf M.M., Azemi S.N., Al-Hadi A.A., Soh P.J., Jamlos M.F. (2017). Dual Band Planar Inverted F Antenna (PIFA) with L-Shape Configuration. MATEC Web Conf..

[20] Wang H., Zheng M. A Triple-Band WLAN Monopole Antenna. Proceedings of the Second European Conference on Antennas and Propagation, EuCAP 2007.

[21] Wang H., Zheng M., Zhang S., Johnson A. Monopole Slot Antenna. United States Patent US 661.

[22] Zheng M., Wang H., Hao Y. (2012). Internal Hexa-Band Folded Monopole/Dipole/Loop Antenna with Four Resonances for Mobile Device. IEEE Trans. Antenn. Propag..

[23] Rowell C., Lam E.Y. (2021). Mobile-Phone Antenna Design. IEEE Antennas Propag. Mag..

[24] Zhang Z. (2017). Antenna Design for Mobile Devices.

[25] Chen Z. (2007). Antennas for Portable Devices.

[26] Jeong S., Cho B., Kwak Y.S., Byun J., Kim A.S. Design and analysis of multi-band antenna for mobile handset applications. Proceedings of the 2009 IEEE Antennas and Propagation Society International Symposium.

[27] Li Y., Zhang Z., Zheng J., Feng Z. (2011). Compact Heptaband Reconfigurable Loop Antenna for Mobile Handset. IEEE Antennas Wirel. Propag. Lett..

[28] Zuo S., Zhang Z., Xie J. (2014). Design of dual-monopole slots antenna integrated with monopole strip for wireless wide area network mobile handset. IET Microw. Antenna Propag..

[29] Singh D., Singh B. (2020). Investigating the impact of data normalization on classification performance. Appl. Soft. Cmput..

[30] Duffy A., Zhan G. (2020). FSV: State of the art and current research fronts. IEEE Electromagn. Compat. Mag..

[31] Bongiorno J., Mariscotti A. (2022). Uncertainty and Sensitivity of the Feature Selective Validation (FSV) Method. Electronics..

[32] 3rd Generation Partnership Project (3GPP) (2024). Evolved Universal Terrestrial Radio Access (E-UTRA). User Equipment (UE) Radio Transmission and Reception.

[33] Li C., Xu C., Wang R., Wu T. (2019). Numerical evaluation of human exposure to 3. 5-GHz electromagnetic field by considering the 3GPP-like channel features. Ann. Telecommun..

[34] Yuan X. (2024). The Case for Electromagnetic Simulation in Mobile Antenna Design.

[35] Cenci M.P., Eidelwein E.M., Scarazzato T., Veit H.M. (2024). Assessment of smartphones’ components and materials for a recycling perspective: Tendencies of composition and target elements. J. Mater..

